# Polydisperse methyl β-cyclodextrin–epichlorohydrin polymers: variable contact time ^13^C CP-MAS solid-state NMR characterization

**DOI:** 10.3762/bjoc.11.299

**Published:** 2015-12-30

**Authors:** Isabelle Mallard, Davy Baudelet, Franca Castiglione, Monica Ferro, Walter Panzeri, Enzio Ragg, Andrea Mele

**Affiliations:** 1 ULCO, UCEIV, F-59140 Dunkerque, France; 2 UCLille, EA GRIIOT (4481), Laboratoire de Pharmacochimie, HEI, 13 rue de Toul, F-59046 Lille, France; 3 UFR Pharmacie, EA GRIIOT (4481), Laboratoire de Chimie Analytique, BP 83, F-59006 Lille, France; 4 Dipartimento di Chimica, Materiali ed Ingegneria Chimica ‘G. Natta’ Politecnico di Milano, Piazza L. da Vinci 32, I-20133 Milano, Italy; 5 CNR-ICRM, Via L. Mancinelli 7, I-20131 Milano, Italy; 6 Dipartimento di Scienze Molecolari Agroalimentari, Università di Milano, Via Celoria, 2 I-20133 Milano, Italy

**Keywords:** dynamic cross-polarization, epichlorohydrin, imprinted, insoluble CRYSMEB polymers, solid-state NMR spectroscopy

## Abstract

The polymerization of partially methylated β-cyclodextrin (CRYSMEB) with epichlorohydrin was carried out in the presence of a known amount of toluene as imprinting agent. Three different preparations (D1, D2 and D3) of imprinted polymers were obtained and characterized by solid-state ^13^C NMR spectroscopy under cross-polarization magic angle spinning (CP-MAS) conditions. The polymers were prepared by using the same synthetic conditions but with different molar ratios of imprinting agent/monomer, leading to morphologically equivalent materials but with different absorption properties. The main purpose of the work was to find a suitable spectroscopic descriptor accounting for the different imprinting process in three homogeneous polymeric networks. The polymers were characterized by studying the kinetics of the cross-polarization process. This approach is based on variable contact time CP-MAS spectra, referred to as VCP-MAS. The analysis of the VCP-MAS spectra provided two relaxation parameters: *T*_CH_ (the CP time constant) and *T*_1ρ_ (the proton spin-lattice relaxation time in the rotating frame). The results and the analysis presented in the paper pointed out that *T*_CH_ is sensitive to the imprinting process, showing variations related to the toluene/cyclodextrin molar ratio used for the preparation of the materials. Conversely, the observed values of *T*_1ρ_ did not show dramatic variations with the imprinting protocol, but rather confirmed that the three polymers are morphologically similar. Thus the combined use of *T*_CH_ and *T*_1ρ_ can be helpful for the characterization and fine tuning of imprinted polymeric matrices.

## Introduction

Cyclodextrin polymers are a subject of great interest because of their use in pharmaceutical industry [1–2], analytical chemistry [3–5], wastewater treatment [6] and food industry [7–9]. Water-insoluble β-cyclodextrin (β-CD) polymers [10] have been widely described to remove organic pollutants [6,11–13] and heavy metals [14] from water. The most efficient method for the synthesis of insoluble polymers is to use di- or polyfunctional linkers with monomers of cyclodextrins. Different effective crosslinkers have been reported in the literature such as epichlorohydrin [15–16], isocyanates [17–18], polycarboxylic acids [19–20] and anhydrides [21]. Following a slightly different approach, Trotta [18] and his group demonstrated that polymerization of cyclodextrins with a variety of synthetic equivalents of di- and tetracarboxylic acids provides an easy, efficient and environmentally sustainable route to highly cross-linked, nanoporous polymers commonly referred to as cyclodextrin nanosponges.

One of the most frequently used crosslinker for insoluble polymers is epichlorohydrin (EP). In this case, the reaction of β-CD with EP requires very strong alkaline conditions to achieve deprotonation of the hydroxyl groups. Then, EP reacts with the alkoxide to form intra- or inter-ether linkages.

We previously proposed the synthesis [22] of soluble and insoluble polymers of a partially secondary rim methylated β-CD (DS = 4.9) commonly called CRYSMEB. In this previous paper, we described the synthesis of imprinted polymers. The imprinting technique is based on interactions between a template and a suitable functional monomer during the prepolymerization process. Once the template is removed, the resulting product is a cross-linked copolymer matrix with specific recognition sites for the template molecule.

As a matter of fact, high-resolution solid-state NMR is a powerful tool to characterize the structure of polymers [23–24] and to study their dynamics [25–26]. Crini has reported previously a solid-state NMR spectroscopy study [27–28] of β-cyclodextrin polymers. At this time, no discussion concerning the NMR spectroscopic characterization of insoluble imprinted CRYSMEB polymers has been reported.

The main purpose of these measurements was to explore possible applications of solid-state NMR spectroscopy for the characterization, at the atomic level, of polymers obtained from polydisperse crystalline methyl β-CD (CRYSMEB) and epichlorohydrin in the presence of a guest molecule. Particular emphasis is devoted to the determination of possible experimental descriptors able to distinguish polymers obtained with the same synthetic route but in the presence of different amounts of imprinting agent, as in the present case. We herein propose an approach based on solid-state ^13^C NMR techniques such as variable contact time cross-polarization magic angle spinning with dipolar decoupling (VCP-MAS) to describe these new materials. The dynamics of cross-polarization can be conveniently exploited to fingerprint the new materials and to provide information for tailored synthesis.

## Results and Discussion

**Scheme 1:**
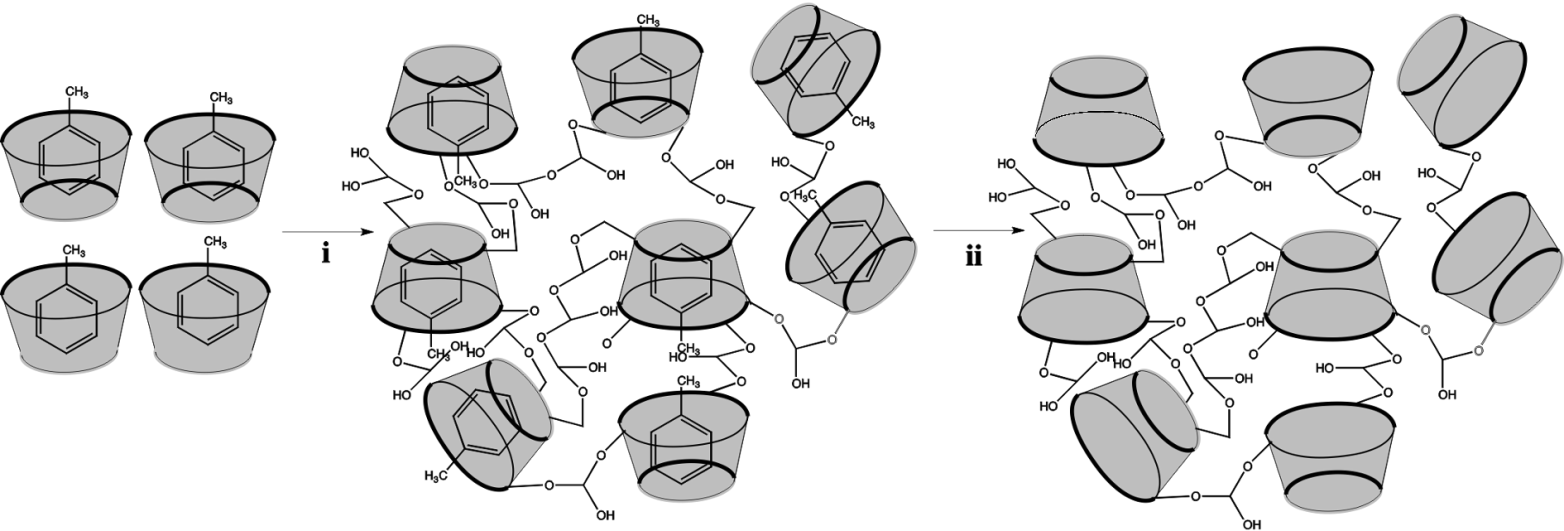
Schematic representation of molecular imprinting technique. i) Polymerization process with toluene-imprinted CRYSMEB and epichlorohydrin, ii) toluene removal.

The molecular imprinting technique allows the introduction of a molecular memory for drugs [29–30] or volatile organic compounds [31–32]. This selective recognition in shape, size and chemical functionality is achieved due to the presence of the target molecule during the polymerization process (Scheme 1).

Initially the target molecules form a complex with the cyclodextrin followed by a copolymerization of the imprinted monomers with the crosslinker. The template introduces highly specific sites into the polymeric network and, after removal of the target the polymer is able to rebind the molecule with high selectivity.

Polymers of CRYSMEB were synthesized according to the procedure of Mallard Favier [22]. Our synthetic protocol was chosen to employ a larger ratio of crosslinker to functional monomer to get insoluble polymers. The deprotonation was carried out in aqueous basic media and imprinting was performed in toluene for 20 min before the polymerization process. After the introduction of toluene, a clear solution was systematically observed. The absence of light diffraction, as expected in the case of toluene aggregation, was controlled by recording the UV–vis absorption spectra in the visible region (400–700 nm), outside the absorption range of toluene. As flattened signals with absorbances close to zero were obtained for all of the mixtures, one can conclude that toluene was dissolved quantitatively in the aqueous phase. Different molar ratios of toluene/CD were used as reported in Table 1. Toluene was chosen as a template for a paradigmatic representative of aromatic organic compounds. The combination of the two parameters such as the value of the binding constant between toluene and β-CD (*K*_f_ = 158 M^−1^) [33] and the high volatility of toluene leads to the conclusion that toluene is a good template for imprinting reactions. According to Ritter [34], the CD–toluene complex fosters mainly the formation of linear polymers instead of globular polymers and the linear polymer network promotes a more efficient guest binding due to a more effective accessibility of the cavity. These points allow an easy removal of toluene by simple drying in the oven under vacuum. Additionally, the control of the polymer growth leading to linear polymer network provided a sufficiently regular system for the interpretation of the NMR results.

**Table 1:** Experimental conditions for the synthesis of polymer.^a^

Polymer	EP/CD	Toluene/CD

D1	40/1	1/4
D2	40/1	4/1
D3	40/1	3/1

^a^Ratios are molar ratios.

Due to the experimental conditions such as high NaOH concentration, high EP/CD ratio and high temperature [15,35], the polymerization process leads exclusively to insoluble cross-linked polymers after EP addition. It should be mentioned that the formation of intralinked bonds between hydroxyl groups and epichlorohydrin was reduced due to the presence of methyl groups in the outer CD cavity. Under the reaction conditions used (33% NaOH, EP/CD= 40 and *T* = 60 °C), gel formation [36] was observed, and the template had no effect on the gel point. Indeed the polymerization process provided insoluble cross-linked polymers without any apparent interference due to the presence of the template during the synthesis.

### FTIR analysis

**Figure 1:**
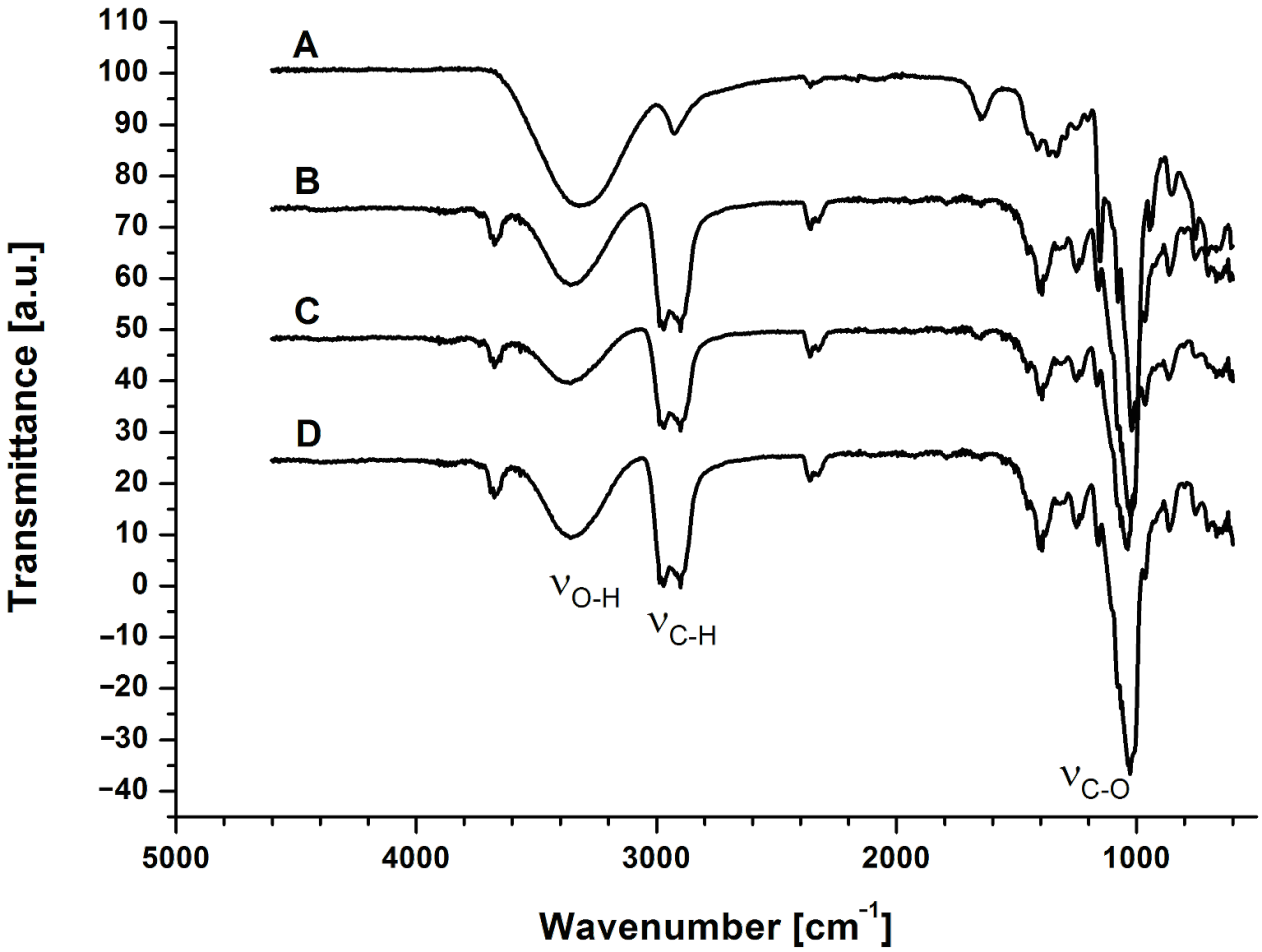
FTIR spectra of (A): native CRYSMEB, (B): D3 (toluene/CD 3:1), (C): D2 (toluene/CD 4:1), and (D): D1 (toluene/CD 1:4) polymers.

A first inspection of the spectroscopic characteristic of D1, D2 and D3 was achieved by FTIR. The infrared spectra of the samples, including reference CRYSMEB, were recorded and are depicted in Figure 1. The fingerprint peaks of the glucopyranose rings were reflected specifically in all polymer spectra: the C–O–C stretching vibration at 1040 cm^−1^, the stretching vibration for the aliphatic CH_2_ at 2900 cm^−1^ and the OH stretching vibration between 3700 and 3000 cm^−1^. As a consequence of the crosslinking process with EP, the spectra of D1, D2 and D3 exhibited a new stretching vibration assigned to CH_2_ groups at 2970 cm^−1^, and a scissoring bending vibration at 1400 cm^−1^. The presence of these more intense bands indicates that EP reacted with the hydroxyl groups. The characteristic water vibration band at around 1650 cm^−1^, assigned to the δHOH bending mode, present in the CRYSMEB spectrum, was indeed absent in the spectra of the polymers. All spectral features observed for the polymers D1, D2 and D3 were in agreement with the results obtained by Orpecio and Evans [37] for β-cyclodextrin–epichlorohydrin polymers.

### Powder X-ray diffraction spectra

**Figure 2:**
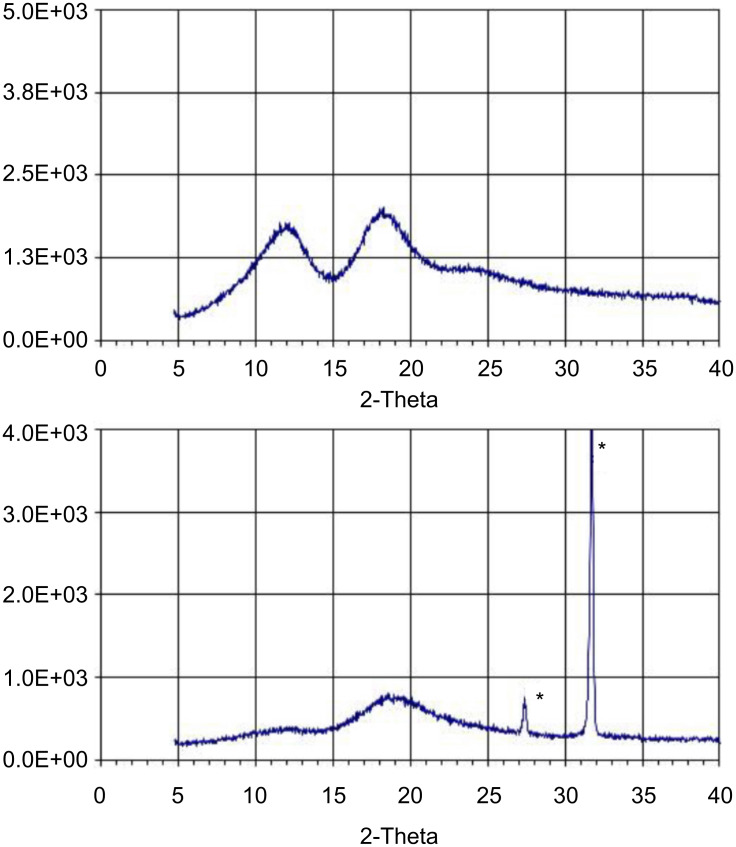
Top: XRPD pattern for CRYSMEB. Bottom: XRPD pattern for D1 polymer. The diffraction peaks denoted with an asterisk at 2θ = 27.48° and 31.7° are due to the presence of traces of residual NaCl. The assignment was confirmed by collecting the XRDP in the 4–100° range of 2θ and comparing the experimental profile with the literature pattern of pure NaCl.

The starting monomer, CRYSMEB and all polymers D1, D2 and D3 were analyzed by X-ray powder diffraction. Figure 2 shows, as an example, the X-ray powder diffraction (XRPD) profiles of CRYSMEB and polymer D1. The upper trace clearly displays the typical halos of the amorphous material. This finding was indeed unexpected, as the starting material was supposed to show non-negligible crystallinity. Similar patterns are observed for D1, as shown in the bottom trace. The diffraction profiles of D2 and D3 were very similar to that obtained for D1 and are not reported here. In all the cases the polymeric material was completely amorphous. However, all the XRPD of D1–D3 samples show a shape of the amorphous profile modified with respect to that of pure CRYSMEB.

### NMR analysis

**Figure 3:**
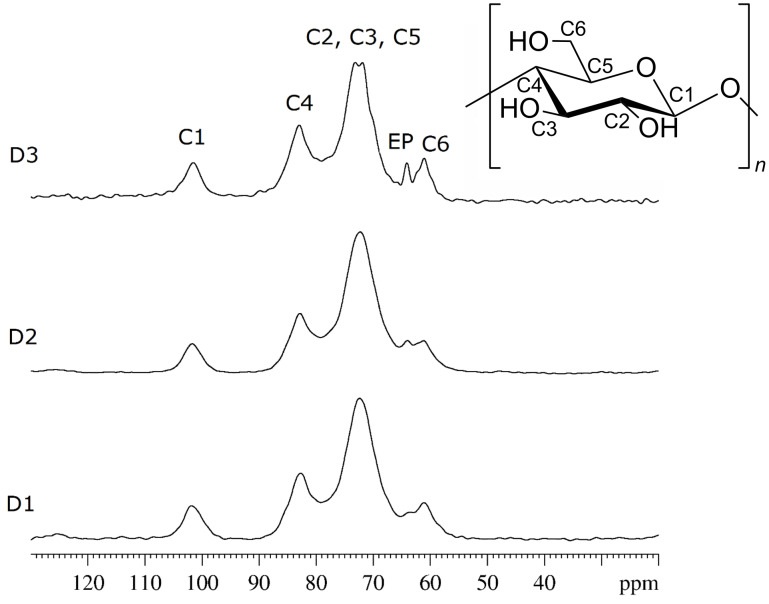
^13^C {^1^H} CP-MAS spectra of polymers D1, D2 and D3. Peak assignment is given in the upper trace.

NMR analysis was carried out by ^13^C {^1^H} solid-state CP-MAS NMR techniques and the experimental spectra of the three samples are shown in Figure 3. The chemical shift range is 50–110 ppm, which is characteristic for the β-CD moiety. The assignment of the polymer resonances is based on the reported values for the crystalline permethylated β-CD. Four carbon resonances due to the glucose unit are observed for all samples and are reported from larger to smaller chemical shift values: C(1), C(4), [C(5), C(3) and C(2)], and C(6). The CH_2_–CH signals of epichlorohydrin are overlapped with the β-CD resonances, and only the signal at 64 ppm can be assigned to the epichlorohydrin moiety [22]. In general all resonances mainly appear as broad single peaks.

No chemical shift changes were observed in the spectra for D1, D2 and D3, indicating that no major structural modifications occurred when the amount of the guest molecule toluene is varied. The line width of the spectral bands in the ^13^C CP-MAS NMR spectra can be qualitatively associated with the crystallinity of the samples: the more crystalline the sample is the sharper are the peaks. However, in the present case the XRPD analysis (vide supra) ruled out any crystalline character of the polymers. Thus, the higher resolution obtained for the sample D3 with respect to D2 and D1 (see top trace in Figure 3), represents an empirical finding. Indeed, all signals in the spectrum of sample D3 are sharper than the corresponding peaks in the spectra of the other two samples, as can be seen considering the peak at 64 ppm which is assigned to epichlorohydrin.

**Figure 4:**
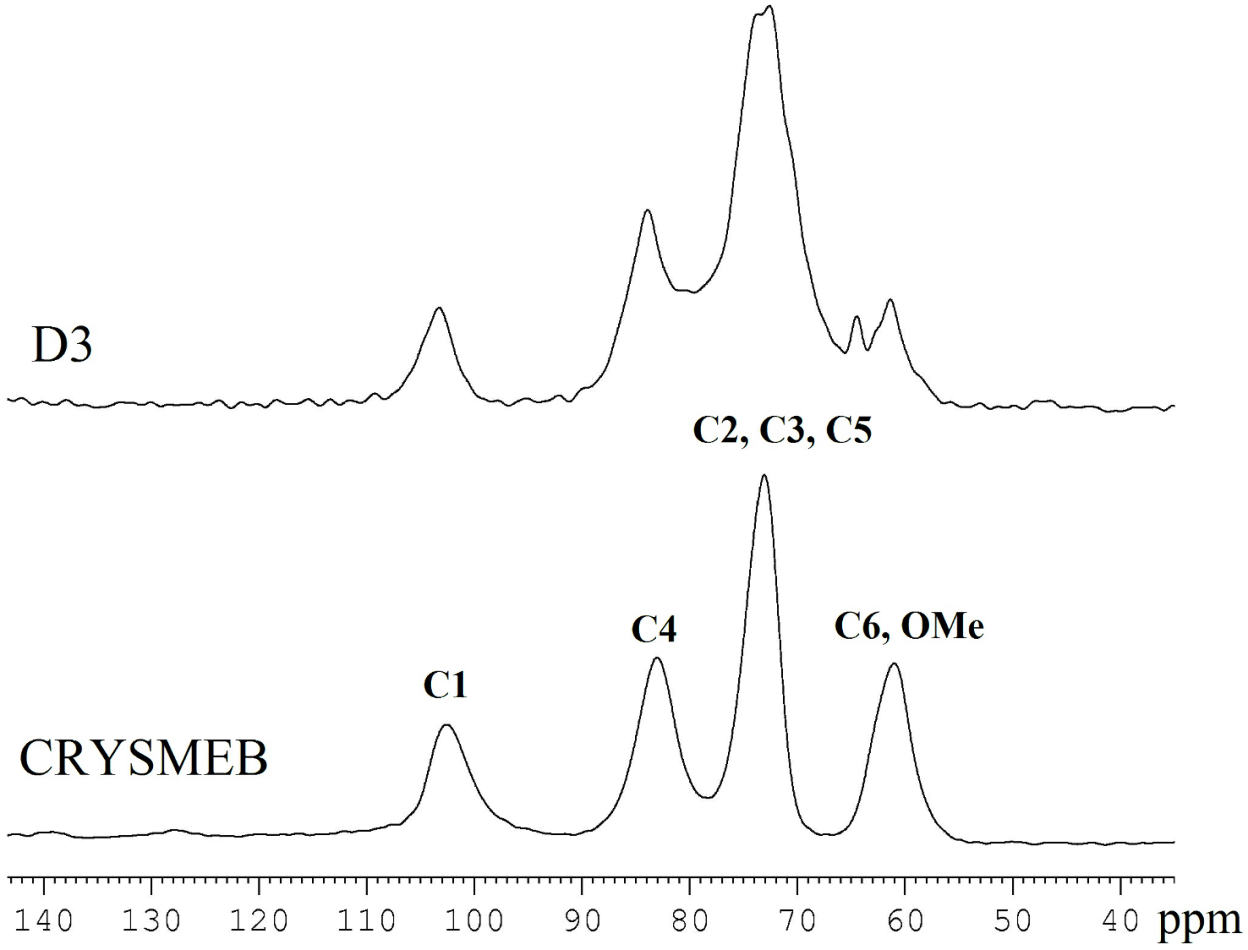
^13^C {^1^H} CP-MAS spectra of native CRYSMEB and polymer D3. Peak assignment is given on CRYSMEB spectrum.

For comparison, a sample of native CRYSMEB was also analyzed. The ^13^C {^1^H} CP-MAS spectrum is reported in Figure 4 together with the spectrum of the D3 polymer. The comparison of the two experimental spectra reveals that no chemical shift variations are observed. Consequently, no relevant structural modification occurs on the β-CD moiety during the polymerization process.

### Dynamics of cross polarization – theoretical description

Important features of the dynamic behavior of a material can be extracted from solid-state NMR data by the analysis of the dynamics of the cross-polarization process.

Cross-polarization from abundant spins ^1^H to dilute spins ^13^C is a double resonance technique mainly used to improve the lower ^13^C sensitivity. If the nuclei are close in space, some dipolar magnetic interactions are established. The nuclei coupled in such a way may transfer polarization from the abundant to the dilute spin (cross-polarization, CP) provided the so called Hartmann–Hahn matching condition if fulfilled. Under CP conditions, a significant sensitivity enhancement of the dilute spin (^13^C) is achieved. The efficiency of CP depends on structural and dynamic factors, related to the sample under investigation. In particular, the internuclear distances and the dynamics of the functional groups are factors affecting the process. Thus, CP provides both structural information (the chemical connectivity) and dynamic insights (the overall molecular dynamics in the solid state). Pines et al. [38] were the first to discuss the effect of different types of motion – such as molecular conformational changes, molecular reorientation and macroscopic sample rotation – on the CP process. Later, the influence of molecular motion on the heteronuclear polarization rate was investigated in several studies [39].

The dynamics of cross-polarization can be explored by performing several experiments with increasing CP contact time and usually it is theoretically described in the ‘fast regime approximation’ according to the thermodynamic model developed by Mehring [40]. At the early stage of CP, the ^13^C magnetization is polarized, arising from the ^1^H–^13^C heteronuclear dipolar interactions through ^1^H reservoirs. Here the growth of the spin magnetization is governed by the cross-polarization rate constant, *T*_CH_^−1^. At long contact times, the ^13^C magnetization follows an exponential decay described by the proton relaxation time in a rotating frame, *T*_1ρ_. The combination of these factors leads to the general law for signal intensity reported in Equation 1:

[1]
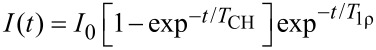


Equation 1 describes the time evolution of the CP intensity *I(t)* as a function of the contact time *t* [38]. The intensity behavior is dictated by two different time constants: *T*_CH_ and *T*_1ρ_, the former affecting the initial part of the curve, the latter driving the final decay. When the two relaxation times deviate by at least two orders of magnitude, it is possible to resolve *T*_1ρ_ and *T*_CH_ parameters separately from the logarithmic plot of the CP intensity against the contact time.

The CP rate constant *T*_CH_^−1^ can be easily extracted from the build-up curves and contains structural and dynamical information. This parameter mainly depends on the number of H atoms attached to a given C atom and on the mobility of the functional group. Thus, a fast cross-polarization process is generally detected in systems containing many H atoms in the proximity of the observed ^13^C nucleus due to the strong dipolar H–C interaction. Additionally, the molecular motion influences the CP rate as well, thus allowing information about molecules that are entrapped or confined within a porous system. In summary, both the H–C distances and the flexibility of the functional groups contribute to the rate of cross-polarization.

### Dynamics of cross polarization – experimental results

**Figure 5:**
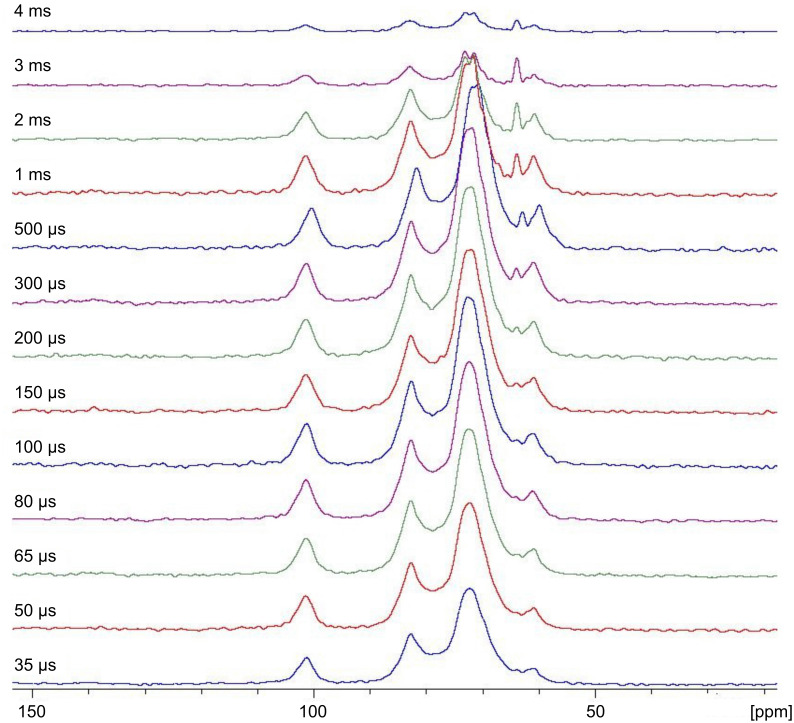
1D ^13^C CP/MAS spectra of polymer D3 as a function of the contact time varying from 35 μs to 4 ms.

The CP magnetization transfer build-up curves were monitored using a constant CP level at various contact times *t*, ranging from 35 μs up to 9 ms. The one-dimensional ^13^C CP-MAS spectra were acquired at the constant MAS rate of 10 kHz for all samples D1, D2, D3 (the spectra of D3 is shown in Figure 5), and native CRYSMEB to test the applicability of this measurement to the samples.

**Figure 6:**
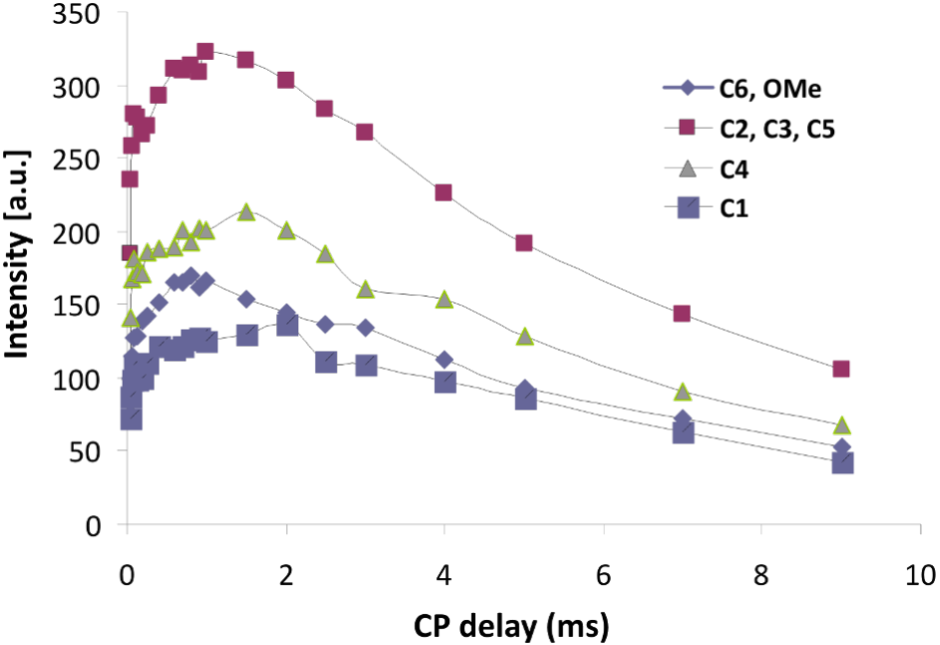
Cross-polymerization (CP) build-up curve of the ^13^C resonances with variable contact times for the CRYSMEB sample.

**Figure 7:**
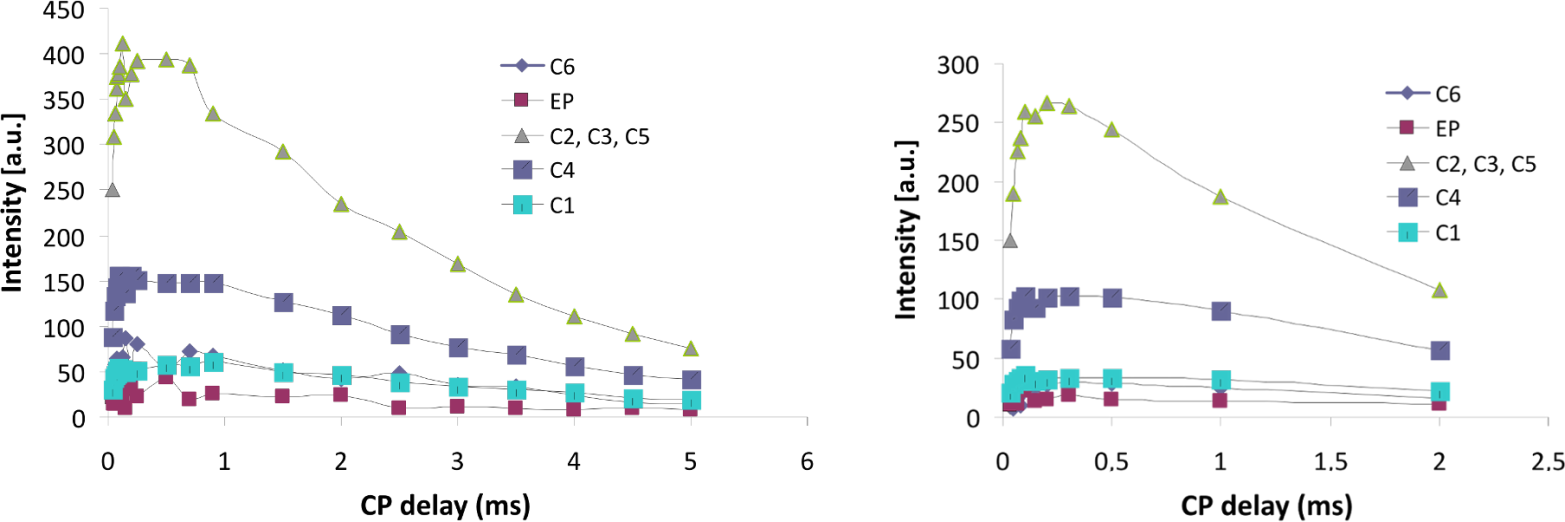
Cross-polymerization (CP) build-up curve of the ^13^C resonances with variable contact time for polymers D1 (left) and D3 (right).

The CP build-up curves for all carbon atoms of CRYSMEB are reported in Figure 6 and the results observed for the polymers D1 and D3 are shown in Figure 7 as an example. In all cases the typical CP dynamics profile was obtained, where the short-time exponential rise of the curves is put down to the ^1^H–^13^C polarization-transfer process due to the residual carbon–proton dipolar interactions, in turn quantified by *T*_CH_. On the other hand, the intensity decay after reaching the saturation level mainly originates from the ^1^H spin–lattice relaxation in the rotating frame (*T*_1ρ_). Both regimes are well-defined within the contact time range chosen in our experiments.

**Figure 8:**
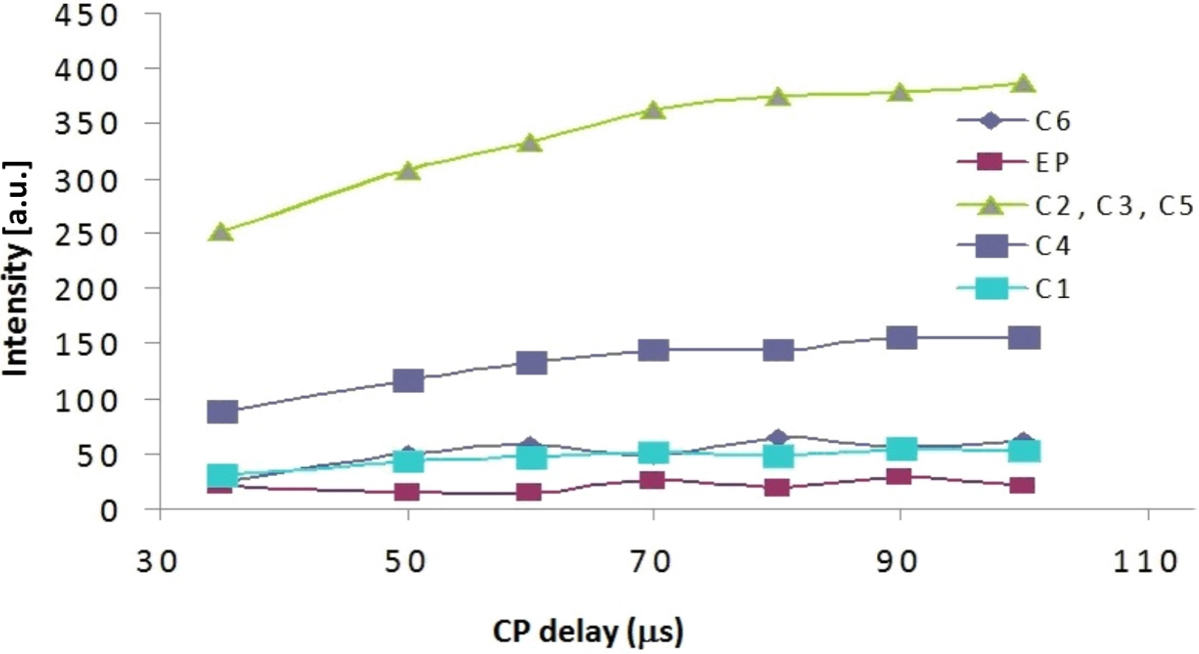
CP build-up curves of the ^13^C resonances with cross-polymerization in the range 0–100 μs for polymer D1.

***T*_CH_ Contact times:** The CP rate (1/*T*_CH_) under spin-locking conditions is determined by the ^1^H and ^13^C relaxation behavior and the effective strength of the dipolar interaction (which is derived from both the C–H distance and molecular motion). The experimental data at short CP times for polymer D1 are illustrated in Figure 8. The plot expanded scale allows to highlight the signals intensities growth in the range of 0–100 μs. A similar behavior is observed for the other polymers D2 and D3.

The maximum of the curve is observed in the range of 0.8–2 ms for the native CRYSMEB sample (see Figure 6), while shorter contact times (<0.9 ms) are observed for all polymers (see Figure 7). This indicates, in the latter cases, a highly efficient magnetization transfer. This is consistent with a rigid molecular frame, as expected in a cross-linked polymer. As a consequence of such molecular stiffness, strong heteronuclear dipolar interactions are established and facilitate an effective magnetization transfer from the proton to various carbon atoms. A good line-shape fitting was obtained for all carbons of the sugar ring (C1–C5) and C6 for CRYSMEB, while for the three polymers the C6 signal is strongly overlapped with the epichlorohydrin resonances, consequently this line was not analyzed. The *T*_CH_ values extracted from fitting the exponential rise of the magnetization build-up curves are listed in Table 2 for all studied samples.

**Table 2:** ^13^C Chemical shifts (ppm), *T*_CH_ values (in μs) and ^1^H spin-lattice relaxation in the rotating frame (*T*_1ρ_ in ms) of the carbon atoms of CRYSMEB and the polymers D1, D2 and D3.

		CRYSMEB		Polymer, D1		Polymer, D2		Polymer, D3	
Peak	Chemical shift (ppm)	*T*_CH_ (μs)	*T*_1ρ_ (ms)	*T*_CH_ (μs)	*T*_1ρ_ (ms)	*T*_CH_ (μs)	*T*_1ρ_ (ms)	*T*_CH_ (μs)	*T*_1ρ_ (ms)

C1	101.4	140.1	6.5	72.4	3.5	160.9	3.6	96.5	3.3
C4	82.7	105.9	6.5	71.2	3.1	173.7	2.9	88.6	2.5
C2, C3, C5	72.3	144.8	6.6	96.5	2.6	160.9	2.4	96.5	1.8
C6	61.1	114.3	6.8	n.d.	3.5	n.d.	1.9	n.d.	2.7
EP	64.2			n.d.	2.5	n.d.	1.7	n.d.	3.6

First of all we propose analyzing the data for comparing the monomer with the three polymers. This can be done by comparing the data present in the columns 3, 5, 7 and 9 of Table 2. For the CRYSMEB moiety all the carbon atoms show similar *T*_CH_ values (Table 2, column 3). Similar results are observed for the three polymers, as shown by the values of columns 5, 7 and 9 in the order. For polymers D1 and D3, the *T*_CH_ values of the β-CD sugar moiety are decreased with respect to that observed for native CRYSMEB, while for polymer D2 the opposite trend is observed. The emerging overall picture is that the polymerization can significantly change the response of the cyclodextrin macro-ring C atoms to the cross-polarization with respect to the pristine CRYSMEB. In detail, the sugar ring C atoms show *T*_CH_ values correlated to the molar ratio of cyclodextrin to toluene. The observed trend *T*_CH_ (D2) > *T*_CH_ (CRYSMEB) > *T*_CH_ (D3) > *T*_CH_ (D1) indicates that the increasing amount of toluene used in the polymerization reaction is likely to generate a progressively more loosely packed polymer network that requires longer contact times for the cross-polarization to be effective. It is also interesting to note that the range of variation of *T*_CH_ values is sufficiently broad to allow for an efficient discrimination among polymers imprinted with close amounts of the imprinting agent. The polymer growth in the presence of increasing amounts of toluene leaves empty voids influencing the cross-polarization rate in terms of average C–H distances, H atoms density in the proximity of the observed C and the local dynamics. The data thus point out that *T*_CH_ can be used as a descriptor to differentiate polymers with the same chemical structure and not showing any spectral differences, as shown by the spectra of Figure 3 where no chemical shift variations can be detected, but indeed differently imprinted by toluene.

***T*_1ρ_ Relaxation times:** Proton *T*_1ρ_ via a resolved carbon resonance provides information on the relative mobility of the H atoms in the molecular frame and whether regions of morphological heterogeneity exist or not. At this stage, it is important to stress two points: i) *T*_1ρ_ describes the relaxation time of the ^1^H nuclei, and thus it is sensitive to hydrogen parameters only, including, as an example, the hydration state. Conversely, the previously reported and discussed *T*_CH_ values depend on the mutual C–H distance and H atoms’ density in the proximity of a given C atom; ii) *T*_1ρ_ and *T*_CH_ span a different time scale, the former in milliseconds, the latter in microseconds.

We have analyzed the magnetization decay for the three polymer samples D1, D2, D3 and the native CRYSMEB. The semi logarithmic plot of *I*/*I_0_* against CP contact time in the range of 1–5 ms shows a linear behavior and 1/*T*_1ρ_ is the slope of the linear fit. The relaxation curves can be represented by a single exponential indicating that any segmental motion inside the polymeric frame can be ruled out. The results are reported in Table 2. All H atoms attached to β-CD carbons of the three polymers exhibit a rapid *T*_1ρ_ relaxation compared to the corresponding H atoms of the native CRYSMEB. This fact is in line with the results previously observed in the case of cyclodextrin nanosponges [41]. A striking feature from the data in Table 2 is that the values of *T*_1ρ_ for all the H atoms of the three polymers are basically the same, with no particular variation related to structure or the synthetic route. In particular, it seems that the imprinting process has no effect on the *T*_1ρ_ relaxation values. These findings thus point out that the three polymers are morphologically homogeneous and do not show conformational heterogeneity. As a consequence, the simultaneous determination of both values *T*_CH_ and *T*_1ρ_ provides complementary information on the effect of the imprinting process and on the overall dynamic behavior of the materials.

## Conclusion

The current study was carried out in order to investigate the structure of insoluble imprinted CRYSMEB polymers using ^13^C solid-state NMR spectroscopy techniques. The proper characterization of these materials in view of their application as selective sorbent for aromatic pollutants necessarily passes through the assessment of the effect of the imprinting process on the polymeric structure. The CP-MAS NMR spectra showed that the three polymers share the same chemical structure, as no apparent chemical shift and line-shape differences are detectable from the ^13^C CP-MAS NMR spectra. The study of the kinetics of the cross-polarization process allowed us to determine, for a large collection of C atoms of all the systems, the *T*_CH_ and *T*_1ρ_ parameters. A clear-cut physical interpretation of the numerical values derived from our experiments requires the formulation of a dynamic model of the polymers, which is not attempted here and, more importantly, not crucial for the main goal of this work: the formulation of an easy and efficient numerical descriptor accounting for the effect of the imprinting agent in the final materials. Indeed, the combined analysis of *T*_CH_ and *T*_1ρ_ provides a fingerprinting discrimination of the three polymeric materials here discussed, D1, D2 and D3. *T*_CH_ variations were found to be nicely associated to the molar ratio of toluene/CRYSMEB (i.e. imprinting agent/monomer), with longer times related to a larger quantity of toluene and, reasonably, to the presence of the imprinted voids in the polymer frame. Conversely, the values of *T*_1ρ_ measured for the H atoms attached to the observed C of the polymers did not reveal any significant variation, either within the same polymer or in comparison with the homologous H atoms of the other polymers. This latter point confirms that the polymer synthesis provides homogeneous materials, without micro-heterogeneities or remarkable morphology changes. We are currently extending this methodology – the combined use of *T*_CH_ and *T*_1ρ_ – in order to provide a general approach for the characterization of the imprinting features of this class of sorbent materials.

CP-MAS NMR spectra of CRYSMEB-EP polymers can be easily collected providing sufficient resolution for assignment of C signals. The exploration of the dynamic behavior and the molecular morphology of the examined systems is, on our opinion, the most innovative and original feature of the investigation. Indeed, the solid-state NMR parameters (i.e. chemical shift, efficiency of cross-polarization and ^1^H *T*_1ρ_ relaxation time) indicate that the addition of toluene leads to a swollen polymer framework and an increase in molecular mobility (as shown by the increased *T*_CH_).

The *T*_CH_ parameter for the various C atoms and for the different batches of polymers could be taken as fingerprint indicator. Moreover, it would be interesting studying a system in which the guest molecules are actually entrapped inside the cross-linked polymer. In this case, the dynamics of the guest could provide precious information on the state of the included molecule inside the polymer network.

## Experimental

### Chemicals

Toluene, sodium hydroxide and epichlorohydrin were purchased from Aldrich and were used as received. CRYSMEB (DS = 4.9) was provided by Roquette Frères (Lestrem, France).

### Materials and instruments

Fourier-transform infrared (FTIR) absorption spectra were recorded using a FTIR Equinox 55 Bruker spectrometer equipped with an ATR module, in the range of 4000–500 cm^−1^ and a resolution of 2 cm^−1^. The dissolution of toluene in the aqueous phase was analyzed by UV–vis spectroscopy (Perkin-Elmer lambda 2S spectrophotometer). X-ray powder diffraction in the 2θ range of 4.7–40° (step size 0.02°; time per step 0.04 s, slits 0.6–8 mm, 30 KV × 10 mA, PSD 3) were collected on a Bruker AXS D2 Phaser diffractometer equipped with LinxEye detector and in Bragg–Brentano geometry, using Cu Kα radiation (λ = 1.54060 Å) with a Ni filter. The data were collected in open air and with a quartz monocrystal zero background sample holder with 0.2 mm depth. Solid-state CP-MAS ^13^C NMR spectra were recorded on a Bruker Avance 600 spectrometer, operating at a frequency of 150.9 MHz and equipped with a MAS probe head. The powder sample was inserted in a 4 mm zirconia rotor and spun in air at 10 kHz speed. The conventional ^13^C spectra were recorded with a proton 90° pulse length of 4 μs, a contact time of 1 ms and 4 s as recycle delay time. Each free induction decay (FID) was acquired with 512 scans and a sweep width of 250 ppm. The TPPM ^1^H decoupling sequence [30] was used during the acquisition period. CP build-up curves were recorded by increasing the length of the contact pulse from 35 μs to 9 ms. This was done in steps of 35 μs at short times, while for times longer than 250 μs the value of the increment was progressively increased. All the experiments were performed at 298 K. The data analysis was performed using the OriginPro software (9.0 version). The NMR spectra were deconvoluted using OriginPro software (9.0 version). Each peak was approximated by a Gaussian function curve fitting analysis.

### General procedure for the preparation of the cyclodextrin polymers

Imprinted polymers were synthesized by a one-step condensation polymerization analogous to that described in [22]. Sodium hydroxide (5 g) was dissolved in water (15 g) and heated under stirring at 60 °C. CRYSMEB (5 g) was added slowly to the solution. Toluene was introduced as template and kept under stirring during 20 min. Then 20 mL of epichlorohydrin were added drop wise to the stirred solution at a rate of about 2 mL every 15 min. At the end of addition, the reaction mixture was kept at 60 °C for 4 h and the polymers were obtained in the gel state. After crushing with ethanol and washing with various solvents, the polymers were dried under vacuum.

## References

[1] Davis M E, Brewster M E (2004). Nat Rev Drug Discovery.

[2] Gidwani B, Vyas A (2014). Colloids Surf, B.

[3] Yu J C, Jiang Z-T, Liu H-Y, Yu J, Zhang L (2003). Anal Chim Acta.

[4] Crini G, Morcellet M (2002). J Sep Sci.

[5] De Klerck K, Mangelings D, Vander Heyden Y (2012). J Pharm Biomed Anal.

[6] Sancey B, Trunfio G, Charles J, Badot P-M, Crini G (2011). J Inclusion Phenom Macrocyclic Chem.

[7] Ciobanu A, Mallard I, Landy A, Brabie G, Nistor D, Fourmentin S (2013). Food Chem.

[8] Ciobanu A, Mallard I, Landy A, Brabie G, Nistor D, Fourmentin S (2012). Carbohydr Polym.

[9] Gazpio C, Sánchez M, Isasi J R, Vélaz I, Martín C, Martínez-Ohárriz C, Zornoza A (2008). Carbohydr Polym.

[10] Morin-Crini N, Crini G (2013). Prog Polym Sci.

[11] Mamba B B, Krause R W, Malefetse T J, Nxumalo E N (2007). Environ Chem Lett.

[12] Mele A, Castiglione F, Malpezzi L, Ganazzoli F, Raffaini G, Trotta F, Rossi B, Fontana A, Giunchi G (2011). J Inclusion Phenom Macrocyclic Chem.

[13] Crini G, Bertini S, Torri G, Naggi A, Sforzini D, Vecchi C, Janus L, Lekchiri Y, Morcellet M (1998). J Appl Polym Sci.

[14] Li N, Wei X, Mei Z, Xiong X, Chen S, Ye M, Ding S (2011). Carbohydr Res.

[15] Renard E, Barnathan G, Deratani A, Sebille B (1997). Macromol Symp.

[16] Vélaz I, Isasi J R, Sánchez M, Uzqueda M, Ponchel G (2007). J Inclusion Phenom Macrocyclic Chem.

[17] Wilson L D, Mohamed M H, Headly J V (2011). J Colloid Interface Sci.

[18] Trotta F, Zanetti M, Cavalli R (2012). Beilstein J Org Chem.

[19] Zhao D, Zhao L, Zhu C, Tian Z, Shen X (2009). Carbohydr Polym.

[20] Zhao D, Zhao L, Zhu C-S, Huang W-Q, Hu J-L (2009). J Inclusion Phenom Macrocyclic Chem.

[21] Girek T, Kozlowski C A, Koziol J J, Walkowiak W, Korus I (2005). Carbohydr Polym.

[22] Mallard Favier I, Baudelet D, Fourmentin S (2011). J Inclusion Phenom Macrocyclic Chem.

[23] Komoroski R A (1986). High Resolution NMR-Spectroscopy of Synthetic Polymers in Bulk.

[24] Ngono-Ravache Y, Foray M-F, Bardet M (2001). Polym Adv Technol.

[25] Bovey F A, Mirau P A (1996). NMR of polymers.

[26] Schmidt-Rohr K, Clauss J, Spiess H W (1992). Macromolecules.

[27] Crini G, Cosentino C, Bertini S, Naggi A, Torri G, Vecchi C, Janus L, Morcellet M (1998). Carbohydr Res.

[28] Crini G, Bourdonneau M, Martel B, Piotto M, Morcellet M, Richert T, Vebrel J, Torri G, Morin N (2000). J Appl Polym Sci.

[29] Deng Y, Cheng C, Quin X, Xian X, Alford T T, Choi H W, Tsow F, Forzani E S (2015). Sens Actuators, B.

[30] Tominaga Y, Kubo T, Yasuda K, Kato K, Hosoya K (2012). Microporous Mesoporous Mater.

[31] Figueiredo L, Erny G L, Santos L, Alves A (2016). Talanta.

[32] Zhang H (2014). Polymer.

[33] Fourmentin S, Ciobanu A, Landy D, Wenz G (2013). Beilstein J Org Chem.

[34] Koopmans C, Ritter H (2008). Macromolecules.

[35] Romo A, Peñas F J, Sevillano X, Isasi J R (2006). J Appl Polym Sci.

[36] Renard E, Deratani A, Volet G, Sebille B (1997). Eur Polym J.

[37] Orprecio R, Evans C H (2003). J Appl Polym Sci.

[38] Pines A, Gibby M G, Waugh J S (1973). J Chem Phys.

[39] Kolodziejski W, Klinowski J (2002). Chem Rev.

[40] Mehring M (1983). Principles of high resolution NMR in solids.

[41] Castiglione F, Crupi V, Majolino D, Mele A, Panzeri W, Rossi B, Trotta F, Venuti V (2013). J Inclusion Phenom Macrocyclic Chem.

